# Modulation of neointimal lesion formation by endogenous androgens is independent of vascular androgen receptor

**DOI:** 10.1093/cvr/cvu142

**Published:** 2014-06-04

**Authors:** Junxi Wu, Patrick W. F. Hadoke, Iris Mair, Win Gel Lim, Eileen Miller, Martin A. Denvir, Lee B. Smith

**Affiliations:** 1 MRC Centre for Reproductive Health, University of Edinburgh, The Queen's Medical Research Institute, 47 Little France Crescent, Edinburgh EH16 4TJ, UK; 2 University/BHF Centre for Cardiovascular Science, University of Edinburgh, The Queen's Medical Research Institute, 47 Little France Crescent, Edinburgh EH16 4TJ, UK

**Keywords:** Androgen receptor, Testosterone, Arterial injury, Neointima

## Abstract

**Aims:**

Low androgen levels have been linked with an increased risk of cardiovascular disease in men. Previous studies have suggested that androgens directly inhibit atherosclerotic lesion formation although the underlying mechanisms for this remain unclear. This study addressed the hypothesis that endogenous androgens inhibit arterial remodelling by a direct action on the androgen receptor (AR) in the vascular wall.

**Methods and results:**

We studied a series of novel mouse lines with cell-specific deletion of the AR in either the endothelium or in smooth muscle cells or both cell types. Findings were compared with a model of global androgen deficiency in wild-type mice (castrated). We characterized the cardiovascular phenotype, vascular pharmacology and histology, and assessed neointimal lesion formation following vascular injury to the femoral artery. Cell-specific AR deletion did not alter body weight, circulating testosterone levels or seminal vesicle weight, but caused limited alterations in arterial contractility and blood pressure. Neointimal lesion formation was unaltered by selective deletion of AR from the vascular endothelium, smooth muscle, or both cell types. Castration in wild-type mice increased neointimal lesion volume (Sham vs. Castration: 2.4 × 10^7^ ± 4.5 × 10^6^ vs. 3.9 × 10^7^ ± 4.9 × 10^6^ µm^3^, *P* = 0.04, *n* = 9–10).

**Conclusion:**

Vascular cell-specific AR deletion had no effect on neointimal lesion formation, while low systemic androgen levels adversely affect neointimal lesion size. These findings suggest that the cardio-protective effects of androgens are mediated either by AR outside the vasculature or by AR-independent mechanisms.

## 1. Introduction

Male sex hormones have traditionally been linked to the greater risk of cardiovascular disease (CVD) in men.^1,2^ However, this view is increasingly being challenged, with considerable recent evidence that testosterone may, in fact, be cardio-protective. Cross-sectional studies have associated *low* testosterone levels with increased cardiovascular risk factors (diabetes mellitus, the metabolic syndrome, abnormal lipid profile) and increased cardiovascular risk in men.^2–5^ This is particularly important given the progressive population-level decline in serum testosterone concentrations in men from developed countries^6–8^ which has resulted in a dramatic increase in the use of androgen replacement therapy (ART). Indeed, there has been a 10-fold increase in prescribed ART in the USA^9^ and nearly a 3-fold increase in the UK^10^ in the past decade. ART improves the muscle/fat mass ratio, bone mineral density, and blood lipid profile^6,11,12^ in hypogonadal men. It has also been suggested that ART could provide a novel strategy to reduce cardiovascular risk. More recently, however, concerns have been raised about safety and the Food and Drug Administration in the USA has announced an investigation into the risk of stroke, heart attack, and death in men taking testosterone products.^13^ This follows recent reports demonstrating an excess of cardiovascular events in apparently hypogonadal men using ART.^14–16^ Given the inconsistent findings from clinical studies, there is a clear need for additional pre-clinical studies to improve our understanding of how endogenous androgens and pharmacological androgen supplements influence CVD. Previous pre-clinical studies have focused on pharmacological testosterone supplementation and/or deficiency (by castration) and have largely supported a cardio-protective role for androgens, with pharmacological testosterone replacement in castrated animals reducing atherosclerotic plaque formation.^17–19^ However, the mechanism of this effect is not clear. It may be indirect, following modification of conventional cardiovascular risk factors, and/or due to direct modulation of vascular remodelling. Furthermore, it has not been established whether androgens alter vascular remodelling by direct stimulation of the androgen receptor (AR), by testosterone-mediated AR-independent actions, or, indirectly, via aromatase-mediated conversion of testosterone to oestrogens. In models of arterial injury that lack elevated systemic cardiovascular risk factors, the findings are contradictory, with studies showing that androgens either reduce^20^ (possibly by inhibiting arterial smooth muscle proliferation),^21^ or have no effect on^22^ neointimal lesion formation.

Endogenous androgens play a complex role in determining cardiovascular risk and thus investigation of their mechanism of action is challenging. The influence of AR stimulation on vascular lesion formation has been investigated previously using the testicular feminised (*Tfm*) mouse, which lacks a functional AR.^19^ However, interpretation of results from this animal is confounded by the fact that it lacks AR in *all* tissues, has low (∼10%) circulating testosterone, and, consequently, has sub-physiological concentrations of oestradiol. Generation of a similar total AR knockout mouse on an atherosclerosis-prone (apoE^−/−^) background suggested that androgens reduce total serum cholesterol via an AR-dependent mechanism but implicated both AR-dependent and AR-independent mechanisms in the observed anti-atherosclerotic effects.^23^ Recognizing the limitations of these models and the complex role of androgens in influencing a number of aspects of cardiovascular risk, we generated mice with vascular cell-specific deletions of AR in order to address the hypothesis that endogenous testosterone inhibits neointimal proliferation by stimulation of AR in the vascular wall.

## 2. Methods

See Supplementary material, Online Data for detailed materials and methods related to this study.

### 2.1 Mice

Animal experiments were performed in accordance both with Directive 2010/63/EU of the European Parliament and with the UK Home Office Animal (Scientific Procedures) Act 1986.

C57Bl/6J mice were supplied by the University of Edinburgh Biomedical Research Facility. Mice with selective ablation of AR from vascular endothelial (VE-ARKO)^24^ or smooth muscle cells (SM-ARKO)^25^ were established in our laboratory as previously described. In this study, these two lines were mated to generate stud males hemizygous for both Tie2-Cre and SM22-Cre, which were then mated with female AR^fl/fl^ mice. The mouse line was maintained by breeding male SM22-Cre^+/−^:Tie2-Cre^+/−^:AR^fl/y^ mice with female AR^fl/fl^ mice. Four genotypes were identified in the resultant offspring at expected Mendelian ratios (∼25% for each genotype in male pups):

1. **WT:** SM22-Cre^−/−^:Tie2-Cre^−/−^:AR^fl/y^. Used as controls.
2. **SM-ARKO:** SM22-Cre^+/−^:Tie2-Cre^−/−^:AR^fl/y^. Smooth muscle cell (SMC) ARKO.
3. **VE-ARKO:** SM22-Cre^−/−^:Tie2-Cre^+/−^:AR^fl/y^. Endothelial cell (EC) ARKO.
4. **SM/VE-ARKO:** SM22-Cre^+/−^:Tie2-Cre^+/−^:AR^fl/y^. Smooth muscle and EC double ARKO.

In this study, only male mice were used for onward analysis.

### 2.2 Determination of genomic ablation of AR and genotyping of mice

To verify AR ablation in target cells, genomic DNA was extracted immediately from freshly isolated aortic EC and SMC cells (without any culturing) and subjected to PCR amplification using primers GCTGATCATAGGCCTCTCTC and TGCCCTGAAAGCAGTCCTCT. An amplicon of 1142 bp indicated the presence of a floxed AR, while an amplicon of 612 bp indicated recombination between loxP sites and deletion of AR exon 2.^24^

Inheritance of Cre Recombinase was used to determine genotype. Genomic DNA from ear clips was amplified using primers CGCATAACCAGTGAAACAGCATTGC and CCCTGTGCTCAGACAGAAATGAGA for Tie2-cre;^26^ and CGCATAACCAGTGAAACAGCATTGC and CAGACACCGAAGCTACTCTCCTTCC for SM22-cre.^27^ An amplicon of 608 bp indicated the inheritance of the Cre Recombinase transgene in EC under control of the Tie2 promoter, while an amplicon of 575 bp for the Cre Recombinase transgene in SMC under control of SM22 promoter.

### 2.3 Vascular cell isolation and culture

Mice were euthanized by CO_2_ and aortic EC and SMC isolated, by collagenase digestion, and cultured, as described.^28^ Isolated cells were either used directly for DNA extraction, or cultured (EC, 7 days in endothelial culture medium; SMC, 14 days in DMEM/F12 GlutaMAX™) for investigation of AR expression. Testosterone (1 × 10^−7^ M), DHT (1 × 10^−8^ M), or vehicle (100% ethanol, 0.1% in final culture medium) was added from the 3rd day of culture and media were replenished twice weekly.

### 2.4 Phenotyping mice with cell-specific AR deletion

#### 2.4.1 Blood pressure measurement

Systolic blood pressure was assessed in conscious, restrained mice using tail-cuff plethysmography (Harvard Apparatus, UK).

#### 2.4.2 Assay for plasma testosterone, total cholesterol, and triglyceride

Plasma testosterone (DEMEDITEC Diagnostics GmbH, Kiel-Wellsee, Germany), total plasma cholesterol (Olympus Diagnostics Ltd, Watford, UK), and plasma triglyceride (Alpha Laboratories Ltd., Eastleigh, UK) were measured using commercially available kits, in accordance with manufacturer's instructions.

#### 2.4.3 Myographic assessment of arterial function

Mice (12–16 weeks) were euthanized by CO_2_. Femoral and mesenteric arteries were isolated for functional analysis, as described previously.^29^ A linear relationship between the increment of cyclic force and the increment of diameter was used to describe arterial compliance.^30^ Arteries were then exposed to high (125 mM) potassium physiological saline solution (KPSS), phenylephrine (PhE, 10^−9^–10^−5^ M), acetylcholine (ACh; 10^−9^–10^−5^ M), and sodium nitroprusside (SNP; 10^−9^–10^−5^ M). A further set of arteries from the same animals were exposed to testosterone (10^−9^–10^−4^ M) and endothelin-1 (ET-1, 10^−11^–10^−7^ M).

### 2.5 Surgical procedures

Surgical procedures were performed in mice under isoflurane-induced anaesthesia with analgesic cover.

### 2.6 Castration

In C57Bl/6J mice, a small incision was made in the mid-line of the scrotum and both testes externalized and removed (castration) or returned to the scrotum (Sham). The mice were allowed to recover for 1 week prior to induction of femoral artery injury.

### 2.7 Femoral artery injury

Wire injury was performed by inserting a guidewire using the method of Sata *et al.*^31^ Ligation injury was performed on the common femoral artery immediately proximal to the femoropopliteal bifurcation. Wounds were sutured and mice were allowed to recover (21 days) to allow lesion development.

### 2.8 Optical projection tomography (OPT)

Mice were killed (sodium pentobarbital) and plasma harvested and stored (−20°C). Mice were then perfusion-fixed, femoral arteries excised from the femoropopliteal branch to the bifurcation with the iliac artery, and then processed for optical projection tomography (OPT), as described.^32^ Longitudinal lesion distribution and total neointimal volume in the first 1.2 mm segment of the artery were used to describe the overall neointima formation (Supplementary material online, *Figure S1*). The maximum cross-sectional neointimal area was determined from serial histological sections indicated the level of stenosis (Supplementary material online, *Figure S1*).

### 2.9 Histology and immuno-fluorescent staining

After OPT scanning, tissues were processed for histology, sectioned (5 µm) and stained with Masson's trichrome. Intimal and luminal area were measured using Image Pro Plus 7.0 rom images obtained using a CoolSNAP camera (Photometrics, UK). Immuno-florescent staining was with Tyramide Signal Amplification (TSA™, PerkinElmer) was applied using primary antibodies against: AR (SantaCruz; 1:400), CD31 (Abcam; 1:300), von Willebrand factor (vWF, Dako; 1:2000), and smooth muscle alpha-actin (SMA, Sigma; 1:1000). Fluorescent images were analysed using confocal microscopy.

For cultured cells, samples were fixed, stained without antigen retrieval, and images were captured using a Zeiss Axiovert 200M epi-fluorescent microscope (Carl Zeiss Ltd., Welwyn, UK).

### 2.10 Statistics

Data are mean ± standard error of the mean (SEM) for *n* mice, unless indicated otherwise. Analysis was performed (GraphPad Prism v5.0) using Student's *t*-test, one-way or two-way ANOVA with a Bonferroni post-hoc test, as appropriate; *P* < 0.05 indicated statistical significance.

## 3. Results

### 3.1 AR localization in vascular tissues

**Figure 1:**
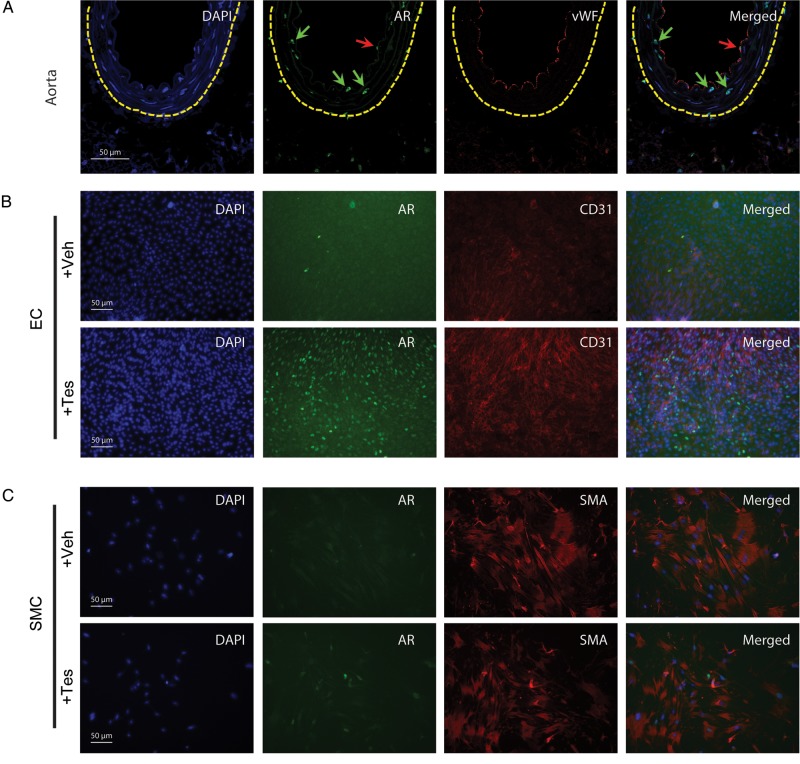
Identification of AR in murine vascular cells. AR is expressed in EC (red arrows) and SMC (green arrows) in healthy mouse aorta (*A*). The dashed yellow line indicates the external elastic lamina. AR expression was up-regulated by testosterone (1 × 10^−7^ M) in cultured mouse aortic EC (*B*) and SMC (*C*). EC were identified using antibodies against vWF and CD31; SMCs were identified using an antibody against SMA. Nuclei were counter-stained with DAPI.

Immunohistochemistry confirmed AR expression in intact mouse aorta (*Figure 1A*) and in cultured aortic EC (*Figure 1B*) and SMC (*Figure 1C*). AR expression in mouse aortic EC and SMC was increased following incubation with testosterone (1 × 10^−7^ M) (*Figure 1B* and *C*).

### 3.2 Establishment of vascular cell-specific AR knockout mice (ARKO)

Three vascular cell-specific AR-ablated mice were generated using the *cre-loxP* system; SM-ARKO (smooth muscle ARKO, generated using SM22-Cre), VE-ARKO (EC ARKO, generated using Tie2-Cre), and SM/VE-ARKO (smooth muscle and ECs, generated from inter-crossing both Cre lines), and floxed-AR mice (WT), which were used as controls.

**Figure 2:**
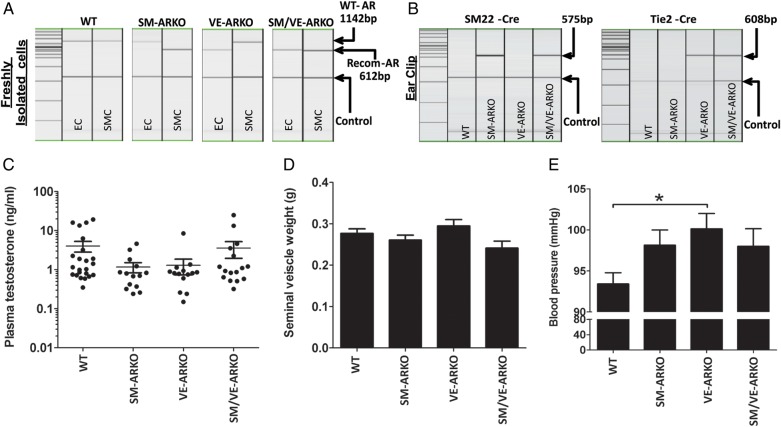
Characterization of mice with vascular cell-specific AR deletion. Cell-specific AR deletion was confirmed (*A*; *n* = 4 for each genotype) using PCR on genomic DNA from freshly isolated aortic EC and SMC. Mouse genotypes were confirmed (*B*) with PCR using genomic DNA from ear clip samples. Deletion of vascular AR did not alter circulating testosterone levels (*C*; *n* = 14–23) or seminal vesicle weight (*D*; *n* = 8–15) but deletion of AR from EC (VE-ARKO) produced a small increase in systolic blood pressure (*E*). *P < 0.05 (*n* = 7–12) by one-way ANOVA plus Bonferroni post-hoc test. (WT = wild-type litter mates carrying floxed-AR; SM-ARKO = AR ablated in SMC, VE-ARKO = AR ablated in EC, SM/VE-ARKO = AR ablated in both EC and SMC.)

In order to confirm deletion of AR in targeted cell types, genomic DNA from freshly isolated aortic EC and SMC were subjected to PCR analysis. An 1142 bp band representing the wild-type AR allele was only observed in WT EC and SMC, SM-ARKO EC and VE-ARKO SMC (*Figure 2A*). A recombined 612 bp band representing the recombined non-functional AR allele was only observed in SM-ARKO SMC, VE-ARKO EC, and SM/VE-ARKO EC/SMC. Genomic DNA samples isolated from ear biopsies were used for genotyping. The SM22-cre amplicon consistently correlated with AR ablation in SMC and the Tie2-cre amplicon with AR ablation in EC (*Figure 2B*).

### 3.3 Characterization of SM-ARKO/VE-ARKO and SM/VE-ARKO mice

Mice of all four genotypes were healthy. In contrast to Tfm or global ARKO mice,^19,23^ SM-ARKO, VE-ARKO, and SM/VE-ARKO mice had normal circulating testosterone concentrations (*Figure 2C*) and seminal vesicle weights (*Figure 2D*). Total plasma cholesterol and triglyceride were not affected by vascular ARKO (Supplementary material online, *Figure S2*). Tail-cuff plethysmography revealed a small but significant increase in blood pressure in VE-ARKO mice (*Figure 2E*).

**Figure 3:**
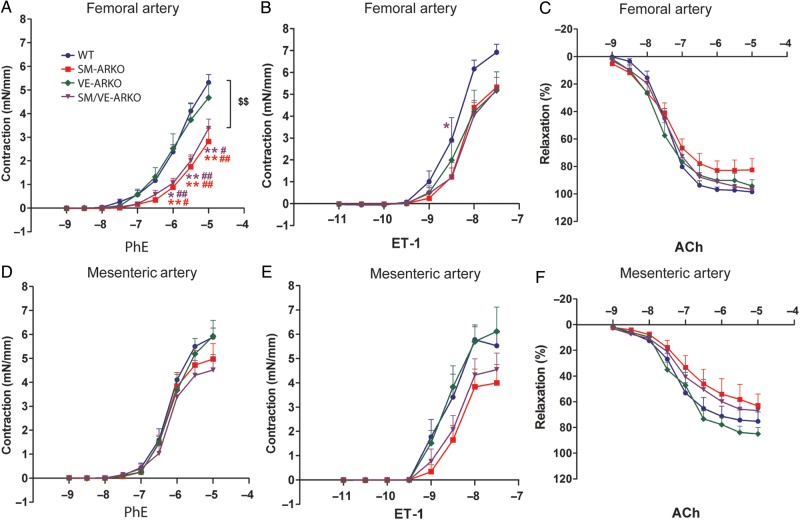
Agonist-dependent vascular dysfunction in mice with selective deletion of vascular AR. In isolated femoral (*A*, *B*, *E*) and mesenteric (*C*, *D*, *F*) arteries cumulative concentration-response curves were produced using phenylephrine (PhE; *A* and *C*) or endothelin-1 (ET-1; *B* and *D*). Acetylcholine (ACh; *E* and *F*) induced-relaxation was obtained after contraction with a sub-maximal concentration of PhE (3 × 10^−6^ M). ^$$^*P* < 0.01 vs. WT; *P < 0.05, **P < 0.01 vs. corresponding WT concentration; ^#^*P* < 0.05, ^##^*P* < 0.01 vs. corresponding VE-ARKO concentration; two-way ANOVA with the Bonferroni post-hoc test. (WT = wild-type litter mates carrying floxed-AR; SM-ARKO = AR ablated in SMC, VE-ARKO = AR ablated in EC, SM/VE-ARKO = AR ablated in both EC and SMC. *n* = 5–9.)

**Figure 4:**
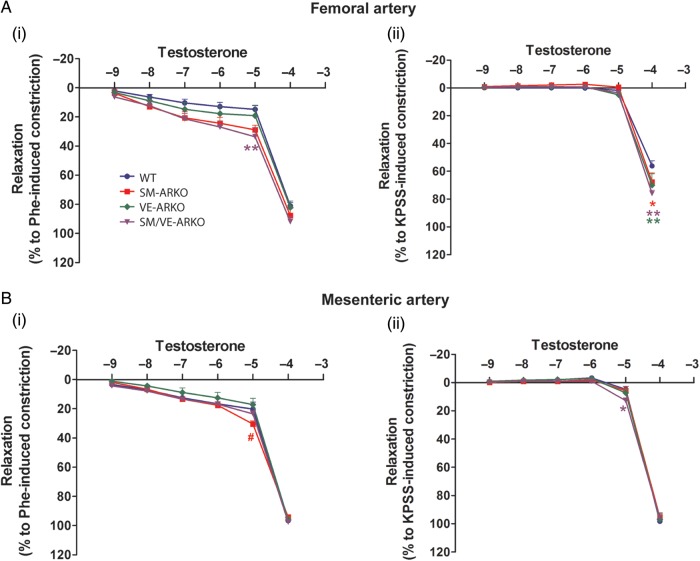
Testosterone induces relaxation in vascular ARKO arteries. Supra-physiological concentration of testosterone induced vascular relaxation both in femoral (*A*) and mesenteric (*B*) arteries from all genotypes, which was independent of the type of pre-constriction. Vascular ARKO produced no dramatic changes in testosterone-mediated relaxation despite some small differences in relaxation at specific concentrations. *P < 0.05, **P < 0.01 vs. corresponding WT concentration; ^#^*P* < 0.05 vs. corresponding VE-ARKO concentration; two-way ANOVA plus Bonferroni post-hoc test. (WT = wild-type litter mates carrying floxed-AR; SM-ARKO = AR ablated in SMC, VE-ARKO = AR ablated in EC, SM/VE-ARKO = AR ablated in both EC and SMC. *n* = 7–9.)

*Ex vivo* myography was used to determine whether vascular AR deletion was associated with functional changes in smooth muscle contraction or endothelium-dependent relaxation. Vascular AR deletion did not alter femoral or mesenteric arterial compliance (Supplementary material online, *Figure S3*). PhE-induced contraction, however, was reduced in femoral arteries that lack smooth muscle cell AR from both SM-ARKO and SM/VE-ARKO mice (*Figure 3A*), while there was a small reduction in ET-1 mediated contraction in all vascular ARKOs (*Figure 3B*). Vascular AR ablation did not alter PhE- (*Figure 3C*) or ET-induced (*Figure 3D*) constriction in mesenteric arteries. KPSS-induced receptor-independent constriction (Supplementary material online, *Figure S4**A(i)* and *B(i)*), ACh-induced endothelium-mediated dilation (*Figure 3E* and *F*), and SNP-induced endothelium-independent dilation (Supplementary material online, *Figure S4**A(ii)* and *B(ii)*) were not affected by vascular ARKO. Testosterone-induced dilation, which occurred at supra-physiological concentrations (1 × 10^−4^ M), showed no dramatic alterations following deletion of AR from vascular EC and/or SMC, despite some small differences in response at specific concentrations (*Figure 4*).

### 3.4 Influence of castration on neointimal lesion formation

**Figure 5:**
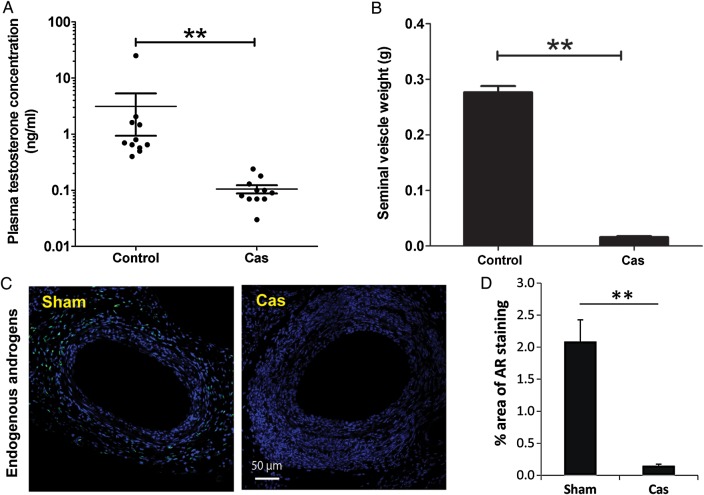
Castration reduces vascular AR expression. Plasma testosterone concentrations (*A*), seminal vesicle weight (*B*), and AR expression in lesion-bearing femoral arteries (*C*) were reduced following castration. **P < 0.01. Data were analysed by Student's *t*-test. (*A*: *n* = 9–11; *B*: *n* = 8–15; *C*: *n* = 9).

**Figure 6:**
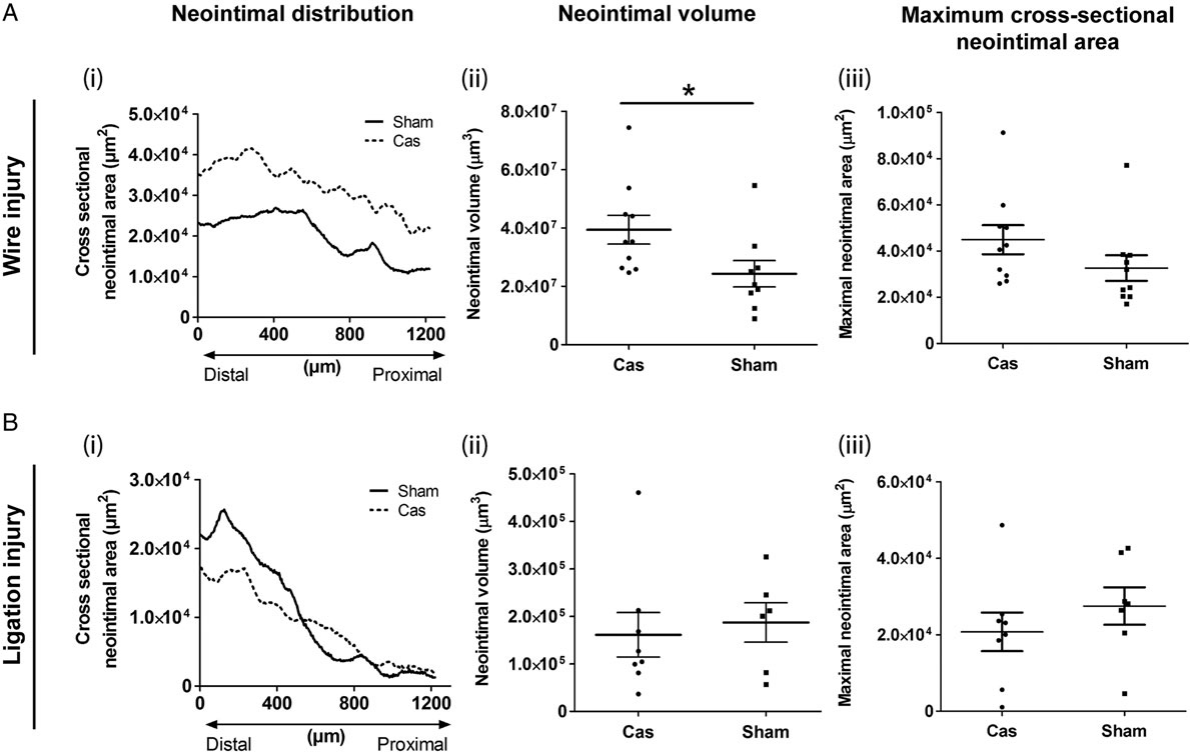
Castration increases neointimal lesion formation following wire injury but not following arterial ligation. In arteries subjected to either (*A*) wire-induced injury or (*B*) ligation, neointimal lesion distribution (i) and neointimal volume (ii) were determined by OPT. Maximal cross-sectional narrowing (iii) was measured in serial sections stained with Masson's trichrome. Panels *A*(i) and *B*(i) show mean neointimal lesion volumes for each group; error bars have been omitted for clarity. Panels *A*(ii & iii) and *B*(ii & iii) show individual data points from each animal in the group with lines and error bars indicating mean ± SEM. *P < 0.05, by Student's *t*-test (*A*: *n* = 8–10; *B*: *n* = 6–8 ).

Castration reduced circulating testosterone concentrations (*Figure 5A*) and seminal vesicle weight (*Figure 5B*) and decreased AR expression in femoral arteries (*Figure 5C*). Body weights following surgery were lower in castrated mice than in controls (Supplementary material online, *Figure S5*). Castration also increased neointimal lesion formation following wire injury (*Figure 6A*), resulting in increased lesion volume but without increasing the maximal cross-sectional area. In contrast, castration had no effect on the neointimal lesion formation following arterial ligation (*Figure 6B*).

### 3.5 Effect of vascular ARKO on neointimal lesion formation

**Figure 7:**
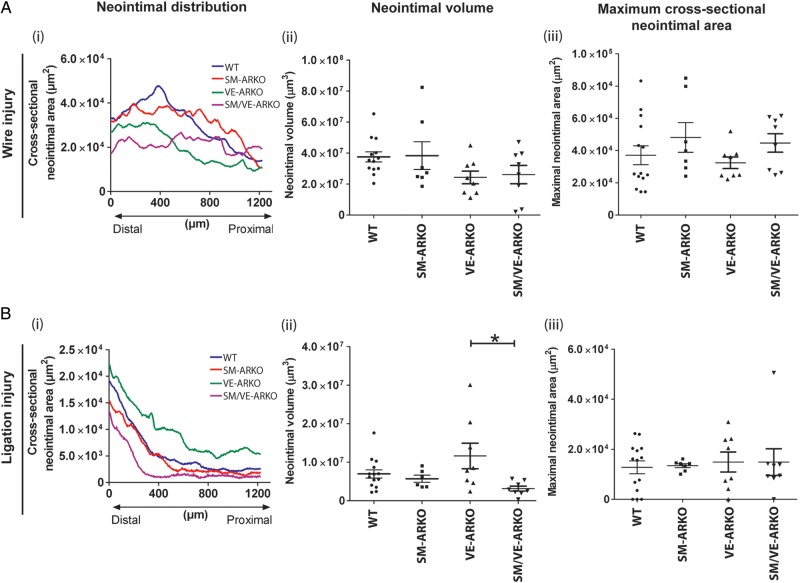
Effect of vascular-specific AR ablation on neointimal lesion formation. Lesion formation following (*A*) wire-induced injury (*n* = 7–14) or (*B*) ligation-induced injury (*n* = 6–14) was determined by optical projection tomography (OPT) and histology. Panels *A*(i) and *B*(i) show mean neointimal lesion volumes for each genotype; error bars have been omitted for clarity. Panels *A*(ii and iii) an *B*(ii and iii) show individual data points from each animal with lines and error bars indicating mean ± SEM. Vascular AR deletion had no effect on lesion formation in response to wire-induced injury. Selective deletion of AR from ECs produced a small increase in neointimal lesion volume following ligation injury (*P < 0.05 by one-way ANOVA plus Bonferroni post-hoc test) but did not alter maximal cross-sectional narrowing. (WT = wild-type litter mates carrying floxed-AR; SM-ARKO = AR ablated in SMC, VE-ARKO = AR ablated in EC, SM/VE-ARKO = AR ablated in both EC and SMC.)

Body-weight changes after arterial injury were similar among the four genotypes of mice (Supplementary material online, *Figure S6*). Vascular ARKO did not alter the profile (*Figure 7A(i)*), volume (*Figure 7A(ii)*), or cross-sectional area (*Figure 7A(iii)*) of lesions induced following wire injury. Similarly, SM-ARKO and SM/VE-ARKO had no effect on lesion size following arterial ligation (*Figure 7B*). In contrast, deletion of AR from vascular ECs alone (VE-ARKO) resulted in an altered lesion profile (*Figure 7B(i)*) and a small increase in lesion volume (*Figure 7B(ii)*), but not cross-sectional area (*Figure 7B(iii)*), following ligation.

## 4. Discussion

Declining testosterone levels in men, combined with increased ART, may both have an impact on the development of CVD possibly by affecting the development of atherosclerotic lesions. Precisely how endogenous androgens influence the formation of vascular lesions remains unknown. This investigation addressed the hypothesis that androgen-induced stimulation of AR in the vascular wall inhibits neontimal lesion development. This was addressed using a series of novel vascular-cell-specific mouse lines with AR deleted from EC and/or SMC. Cell-specific deletion of vascular AR did not alter neointimal lesion formation, while castration in wild-type mice did result in adverse neointimal lesion formation. These contrasting findings suggest that the effects of androgens on vascular lesion formation are mediated by one or more of the following mechanisms: (i) activation of AR-independent pathways, (ii) secondary to aromatization of androgen to oestrogen, (iii) stimulation of AR in other cell types.

The generation of vascular-specific ARKO mice was central to this investigation. The functional role of AR has been investigated previously using *Tfm*^19^ mice. Interpretation of data from these animals is complicated, however, by low endogenous levels of testosterone and oestradiol (requiring pharmacological replacement). In contrast, consistent with previous demonstrations,^22,23^ vascular cell-specific deletion of AR did not reduce circulating testosterone levels and, therefore, no exogenous androgen administration was required. This is important since pharmacological administration of androgens does not satisfactorily ‘replace’ endogenous hormone. The pharmacokinetics and tissue accumulation of pharmacologically administered androgen are not fully understood (thus overdosing may occur if the serum/plasma testosterone level is the only clinical parameter for androgen prescription). This may explain (i) the unexpected increase in adverse cardiovascular events associated with androgen treated in a clinical trial involving elderly (>65 years old) hypogonadal men,^16^ and (ii) the increased death and incidence of stroke and myocardial infarction (regardless of the pre-existing CVD) associated with clinical androgen replacement in hypogonadal men, as demonstrated in a large scale observational study.^14^

Androgens contribute to elevation of blood pressure by acting on catecholamines in the brain, independent of classical AR; blood pressure in wild-type and *Tfm* rats is reduced by castration and restored by administration of testosterone.^33^ This contrasts with vascular selective ARKO which showed either no change or, in the case of VE-ARKO, a small elevation in blood pressure. The implication, therefore, is that testosterone-mediated elevation of blood pressure is not mediated by vascular AR. The increased blood pressure in VE-ARKO mice suggests slight EC dysfunction, but this was not supported by our findings in isolated arteries.

Disruption of systemic androgen/AR signalling impairs normal vascular function.^34^ In *Tfm* mice, contraction in response to high potassium (but not to noradrenaline) was reduced in femoral arteries, suggesting altered smooth muscle cell function.^34^ Relaxation in response to ACh was also impaired, suggesting EC dysfunction.^34^ Vascular AR ablation had no effect on passive arterial compliance and did not alter endothelium-dependent or -independent relaxation, confirming normal activity of the endothelium-derived nitric oxide system. It was notable that EC function was maintained in VE-ARKO mice, despite the (small) increase in blood pressure in these animals. Unlike the *Tfm*, smooth muscle AR deletion impaired agonist-mediated contraction without altering the response to potassium, suggesting a change in receptor-dependent signal transduction pathways. Reduced contraction was agonist dependent (being more evident in response to noradrenaline than to ET-1) and tissue-specific (more obvious in the femoral, than in the mesenteric, artery). An androgen-mediated alteration of adrenoceptor activation would be consistent with androgen-induced production and release of noradrenaline in rodents.^35^ The differences between the current results and those reported for *Tfm* suggest that AR expressed outside the vasculature contributes to regulation of vascular function. The low testosterone in *Tfm* could also lead to a loss of rapid, non-genomic androgen signalling. Alternatively, since endogenous oestrogen regulates EC function in males,^36,37^ the impaired endothelium-mediated dilation in the *Tfm* mice could be a result of its low oestrogen levels.

As shown previously,^38^ testosterone caused relaxation of arteries, although only at supra-physiological concentrations. The maintenance of this response in the vascular-specific ARKO mice is consistent with data from *Tfm* mice.^34^ This indicates, therefore, that vascular AR does not mediate this response. Similar arterial relaxation has been observed with high concentrations (≥1 × 10^−4^ M) of corticosteroids, oestrogen, and cholesterol^39,40^ and may be due to direct alteration of the cell membrane. Given the very high concentrations required to produce this response, testosterone-induced vasodilation is very unlikely to have any physiological relevance.

Evidence for androgen-mediated inhibition of vascular neointimal lesion formation under normolipidemic conditions is less conclusive than for inhibition of atherosclerosis.^20,22^ This investigation addressed the influence of androgens/AR on neointimal lesion formation using models of denuding (wire) and non-denuding (ligation)^41^ injury. Wire injury denudes the endothelium and produces lesions formed predominantly from circulating bone marrow-derived progenitor cells. On the other hand, femoral artery ligation does not introduce direct endothelial damage. The disturbed blood flow and altered shear stress may induce endothelial dysfunction and stimulate neointima formation by migration and proliferation of media-derived mural smooth muscle cells.^42^ Increased lesion volume in castrated mice following wire-injury, but not following ligation, is consistent with evidence that the influence of androgens is dependent on the type of lesion.^20^ Endothelial denudation is one of the major differences between the wire injury and ligation injury models. Androgens promote endothelial proliferation and migration *in vitro*^43^ and improve angiogenesis *in vivo* following ischaemic injury.^44,45^ It is possible, therefore, that endothelial regeneration following wire injury was impaired by systemic androgen deprivation, thus favouring formation of lesions. In addition, blocking AR signalling improves self-renewal and migration of bone marrow-derived stem cells^46^ which may promote lesion formation following wire injury. The well-recognized androgen/AR mediated immune-suppression^47^ may also modulate adventitial inflammation and subsequent neointima formation and could explain the increased lesion volume following castration.

Following arterial injury, vascular AR ablation had very little impact in neointima formation. Deletion of AR from the endothelium increased lesion volume following arterial ligation, but it was notable that no similar increase was detected in mice with double knockout of AR in EC and SMC. This may suggest that deletion of AR in the smooth muscle opposes the effect of deletion from the endothelium. However, given the variability of the data in the VE-ARKO following ligation, it seems more likely that this result is an anomaly, and that, as proposed, AR in the vascular EC and SMC does not contribute to regulation of neointimal lesion formation. Excluding a role for the EC and SMC AR suggests that any influence of androgens on neointimal lesion formation may be mediated by AR-independent mechanisms, or by conversion to oestrogen by aromatization either systemically or locally in the vascular wall, or by AR expressed in other cell types in the vascular wall. It should be noted, however, that AR expression in the vasculature is not restricted to EC and SMC. In healthy arteries, cells bearing strong AR expression were shown to be present in the adventitia, while the population of AR-positive adventitial cells was increased in injured arteries. It was notable that expression of AR in the adventitia of injured arteries was dramatically reduced following castration. However, the identity of these AR positive cells and their pathophysiological function require further investigation. Interestingly, androgens were recently reported to increase ischaemia-induced angiogenesis, indicating a direct association between androgen and ECs.^43,44^ Our VE-ARKO mice will be a useful tool to address the question whether endothelial AR truly regulates endothelial function or behaviour using endothelial-specific models.

The use of vascular selective ARKO mice has shown that AR in the arterial wall have little role in regulating androgen-dependent neointimal lesion formation. These results suggest that any protective effects of androgens in atherosclerosis are likely to be mediated by conversion of testosterone to oestrogens, by effects on classical cardiovascular risk factors such as cholesterol, or by AR outside the vascular wall. Future investigations should try to determine whether androgens inhibit atherosclerosis through direct modulation of non-vascular AR or following conversion to oestrogens.

## Supplementary material

Supplementary material is available at *Cardiovascular Research* online.

## Funding

Funded by a British Heart Foundation Project Grant award to L.B.S., P.W.F.H., and M.A.D. (PG/11/72/29334), an MRC Programme Grant award to L.B.S. (G1100354), and the BHF Centre for Research Excellence. W.G.L. received a BHF CoRE Summer Studentship. I.M. received a Study-abroad Fellowship of the German National Academic Foundation. Funding to pay the Open Access publication charges for this article was provided by The British Heart Foundation.

## Supplementary Material

Supplementary Data
