# Supplementary material for: Modulation of neointimal lesion formation by endogenous androgens is independent of vascular androgen receptor
Source: Cardiovasc Res. 2014 Jun 4;103(2):281–90. doi: 10.1093/cvr/cvu142 (PMC4094672; doi:10.1093/cvr/cvu142)
Supplement: Supplementary Data [file supp_103_2_281__index.html]

Modulation of neointimal lesion formation by endogenous androgens is independent of vascular androgen receptor — Modulation of neointimal lesion formation by endogenous androgens is independent of vascular androgen receptor — Supplementary Data 

# Modulation of neointimal lesion formation by endogenous androgens is independent of vascular androgen receptor

## Supplementary Data

Supplementary Data

**Files in this Data Supplement:**

- Supplementary Data - Doc file
