# Supplementary material for: Modulation of neointimal lesion formation by endogenous androgens is independent of vascular androgen receptor
Source: Cardiovasc Res. 2014 Jun 4;103(2):281–90. doi: 10.1093/cvr/cvu142 (PMC4094672; doi:10.1093/cvr/cvu142)

# Materials and Methods

**Mice**

Animal experiments were performed in accordance both with Directive 2010/63/EU of the European Parliament and with the UK Home Office Animal (Scientific Procedures) Act 1986, and licenced under Project Licence PPL 60/4523. All procedures passed local ethical review. C57Bl/6J mice were supplied by the University of Edinburgh Biomedical Research Facility. Genetically modified mice in which the AR has been selectively ablated from vascular endothelial cells (VE-ARKO) or smooth muscle cells (SM-ARKO) were generated as described. We inter-crossed these two lines to generate a new line lacking AR from both vascular cell-types (SM/VE-ARKO). Males carrying both SM22-Cre and Tie2-Cre were mated to females homozygous for the X-linked floxed-AR to generate experimental animals. All mice were maintained on a C57Bl/6J background. The male offspring included the four genotypes listed below:

1. **WT:** SM22-Cre^-/-^:Tie2-Cre^-/-^:AR^fl/y^. Used as controls throughout the study.
2. **SM-ARKO:** SM22-Cre^+/-^:Tie2-Cre^-/-^:AR^fl/y^. Smooth muscle cell ARKO.
3. **VE-ARKO:** SM22-Cre^-/-^:Tie2-Cre^+/-^:AR^fl/y^. Endothelial cell ARKO.
4. **SM/VE-ARKO:** SM22-Cre^+/-^:Tie2-Cre^+/-^:AR^fl/y^. Smooth muscle/endothelial cell ARKO.

Mice (3-5/ group) were maintained at 21±2°C, 50% humidity, and 12 hour light/dark cycle with *ad libitum* access to chow and water.

**Determination of genomic ablation of AR and genotyping of mice**

In order to verifying specific ablation of AR gene in targeted cells, genomic DNA was extracted from freshly isolated aortic endothelial cells (EC) and smooth muscle cells (SMC) respectively, and subjected to PCR amplification using primers GCTGATCATAGGCCTCTCTC and TGCCCTGAAAGCAGTCCTCT. PCR amplification products were resolved using a QiaXcel capillary system (Qiagen, UK). An amplicon of 1142 bp indicated presence of a floxed AR whilst an amplicon of 612 bp indicated recombination between loxP sites and deletion of AR exon 2.[^2^](#_ENREF_2)Inheritance of Cre Recombinase was used to determine genotypes of mice. Genomic DNA was extracted from frozen ear clip of each mouse. Primers CGCATAACCAGTGAAACAGCATTGC and CCCTGTGCTCAGACAGAAATGAGA were used for Tie2-cre[^3^](#_ENREF_3); and primers CGCATAACCAGTGAAACAGCATTGC and CAGACACCGAAGCTACTCTCCTTCC for SM22-cre[^4^](#_ENREF_4). PCR amplification products were resolved using a QiaXcel capillary system (Qiagen, UK). An amplicon of 608 bp indicated inheritance of the Cre Recombinase transgene in EC under control of Tie2 promoter, whilst an amplicon of 575 bp for the Cre Recombinase transgene in SMC under control of SM22 promoter.

**Vascular cell isolation and culture**

Mice were euthanized by CO_2_. Aortic EC and SMC were isolated and cultured as described[^5^](#_ENREF_5). Briefly, the mouse thoracic aorta was carefully dissected and incubated with collagenase type II (Sigma-Aldrich, UK; 30min, 37^o^C). The endothelial cells were flushed off with endothelial culture medium (DMEM/F12 GlutaMAX™ (Life Technologies, UK) supplemented with 10% foetal bovine serum (Life Technologies, UK), 1x non-essential amino acids (Sigma-Aldrich, UK), Penicillin/streptomycin (50 units/ml and 50 µg/ml, respectively), endothelial cell growth supplement (3 µg/ml, Sigma-Aldrich, UK), and heparin (20 units/ml, LEO Laboratories Limited, UK)). The adventitia was then peeled off and discarded. The rest of the aortic segment was mainly composed of medial smooth muscle cells, which was further digested with collagenase (3 h, 37^o^C). The isolated cells were either used directly for DNA extraction, or cultured for investigation of AR expression. EC were cultured (7 days) in endothelial culture medium. SMC were cultured (14 days) in DMEM/F12 GlutaMAX™ (Life Technologies, UK) supplemented with 10% foetal bovine serum (Life Technologies, UK). Testosterone (1x10^-7^M), DHT (1x10^-8^M) or vehicle (100% ethanol, 0.1% in final culture medium) were added from the 3^rd^ day of culture. Medium was replaced twice a week with designated drugs or vehicle.

## Myography

Mice (aged 12-16 weeks) were culled by asphyxiation using a rising concentration of CO_2_, and femoral arteries and mesenteric arteries were isolated for functional analysis using small-vessel wire myography (Multi-myograph 610, Danish Myotech, Denmark) as described[^6^](#_ENREF_6). Femoral artery rings (2mm in length) were set to passive tension equivalent to 100 mmHg, and mesenteric artery to 50mmHg. A linear relationship between the increment of cyclic force and the increment of diameter was observed in all artery rings. The slope of the curve was then used to describe the arterial compliance[^7^](#_ENREF_7). Following contraction with high potassium physiological saline solution (KPSS), cumulative concentration-response curves were obtained using phenylephrine (PhE, 10^-9^–10^-5^M), acetylcholine (ACh; 10^-9^–10^-5^M) and sodium nitroprusside (SNP; 10^-9^–10^-5^M). A further set of arterial rings from the same animals were used for testing testosterone (10^-9^–10^-4^M), and endothelin-1 (ET-1, 10^-11^–10^-7^M). Vasodilator responses were obtained after contraction with a sub-maximal concentration of PhE (3x10^-6^M). For testosterone-induced dilation, vessel rings were pre-contracted with PhE (3x10^-6^M) and KPSS, respectively. At the end of each experiment, each arterial ring was contracted with KPSS for 20min to confirm its viability.

## Surgical Procedures

Surgical procedures were performed in mice under general anaesthesia (inhalation of isoflurane; 5% for induction 2-3% for maintenance) with appropriate analgesic cover (buprenophine; 0.05mg/kg body weight, sc). Depth of anaesthesia was indicated by loss of the pedal withdrawal reflex.

**Castration**

Male C57Bl/6J mice were randomly divided into groups receiving castration or sham castration. Briefly, a small incision was made in the mid-line of the scrotum and both testes externalised. For animals undergoing castration testes were removed following ligation of the testicular blood supply, whilst the testes were returned to the scrotum in sham castration mice. The mice were allowed to recover for 1weeks prior to induction of femoral artery injury.

**Femoral artery injury**

In each hind limb, the femoral artery was isolated from the vein and nerve. **Wire-injury** was performed using the method of Sata *et al.*[^8^](#_ENREF_8). Briefly, a 0.015” straight sprung angioplasty guide wire (Cook Inc., USA) was advanced (~1cm) into the femoral artery in the direction of the iliac artery. The wire was then withdrawn and blood flow re-established across injured areas of the femoral artery. **Ligation injury** was performed by isolating and ligating the common femoral artery immediately proximal to the femoropopliteal bifurcation. Wounds were sutured (6-0 Mersilk) and mice were allowed to recover for 21 days to allow neointimal lesion development.

## Blood pressure measurement

Systolic blood pressure was assessed using tail cuff plethysmography (Harvard Apparatus, UK). Mice were trained on the procedure before data acquisition was started. For each mouse, the blood pressure was presented as the mean value of 4 consecutive measurements on the first day and a further four measurements three days later.

**Assay for plasma testosterone, total cholesterol and triglyceride**

Plasma testosterone level was analyzed using a commercial mouse testosterone ELISA Kit (DEMEDITEC Diagnostics GmbH, Kiel-Wellsee, Germany) according to the manufacturer's instructions. Total plasma cholesterol and triglyceride measurements were determined using commercial kits (Olympus Diagnostics Ltd, Watford, UK and Alpha Laboratories Ltd., Eastleigh, UK, respectively) adapted for use on a Cobas Fara centrifugal analyzer (Roche Diagnostics Ltd, Welwyn Garden City, UK).

## Optical Projection Tomography (OPT)

Three weeks after femoral artery injury, mice were killed by lethal dose of sodium pentobarbital. Blood was collected from the abdominal vena cava into a heparinized syringe. Plasma was harvested via centrifugation of whole blood donations and stored at -20^o^C for future tests. Mice were then perfusion-fixed with 10% neutral buffered formalin (Sigma-Aldrich, UK). Femoral arteries were excised from the femoropopliteal branch to the bifurcation with the iliac artery (thereby including a proximal non-injured segment). Fixed arteries were processed for optical projection tomography (OPT) as described[^9^](#_ENREF_9). Briefly, arteries were embedded in filtered 1.5% low melting point agarose, dehydrated in absolute methanol (24h) and then optically-cleared in 1:2 v/v benzyl alcohol: benzyl benzoate (BABB). Arteries were imaged using a Bioptonics 3001 OPT tomograph (SkyScan, UK). Tomographic 3D images were generated using Nrecon software (SkyScan, UK) and data analyzed using CTAn software (SkyScan, UK). The longitudinal neointima distribution and total neointimal volume of the first 1.2mm segment of the injured artery were used to describe the overall neointima formation, and the maximum cross-sectional neointimal area obtained from serial histological sections indicated the level of stenosis (Suppl Figure 1).

## Histology and Immuno-fluorescent staining

After OPT scanning, agarose blocks were processed for histology and embedded in paraffin. Sections (5µm) of lesion-containing artery were stained with Masson’s trichrome using a standard protocol. Images were digitized using a CoolSNAP camera (photometrics, UK) and intimal and luminal area were measured using Image Pro Plus 7.0. Immuno-florescent staining was following antigen retrieval, blockade of endogenous peroxidase activity (3% H_2_O_2_) and non-specific binding (in 10% normal goat serum and 5% BSA), and then treatment with the appropriate primary antibody followed by a complementary secondary antibody conjugated with either fluorescent dye or horse radish peroxidase (HRP) which was further visualized with Tyramide Signal Amplification (TSA™, PerkinElmer)[^1^](#_ENREF_1). The primary antibodies used were: AR (SantaCruz; 1:400), CD31 (Abcam; 1:300), von Willebrand factor (vWF, Dako; 1:2000), smooth muscle alpha-actin (SMA, Sigma; 1:1000). Fluorescent images for tissue sections were captured using a Zeiss LSM 510 Meta Axiovert 100M confocal microscope (Carl Zeiss Ltd., Welwyn, UK). For cultured cells, samples were fixed with cold methanol for 10 minutes and stained without antigen retrieval. Cell images were captured using a Zeiss Axiovert 200M epi-fluorescent microscope (Carl Zeiss Ltd., Welwyn, UK).

## Statistics

All data are expressed as mean ± standard error of the mean (SEM) where n refers to the number of mice. Data between two groups were analyzed using Student’s t-test. Data from multiple groups were analyzed using one-way or two-way ANOVA with a Bonferroni post-hoc test, as appropriate. Analyses were performed using GraphPad Prism v5.0. Differences were considered statistically significant when p<0.05.

**Results**

**Supplemental Figure 1. Lesion analysis using Optical projection tomography and histology.** Injured femoral arteries were fixed, embedded 1.5% agarose, dehydrated and clearer. The entire sample was then scanned by OPT (0.9 degree/step for 360 degrees) to produce 400 longitudinal images (2^nd^ panel). The images were reconstituted to produce 1022 serial cross-sectional images (3^rd^ panel) using NRecon software (SkyScan, UK). The neointimal area on each cross-sectional image was quantified (CTan software; SkyScan, UK), allowing quantification of the longtitudinal distribution of the lesion (top panel) as well as the neointimal volume in given length of arterial segment. In most injured arteries lesion formation was most extensive within ~1mm of the wire insertion point or the site of ligation. Thus the first 300 serial sections (about 1.2mm in length @ 4.064µm/pixel) were used for data analysis (between the dashed lines). After OPT scanning, the vessel was processed for histological examination. Serial sections by Masson’s tri-chrome staining matched closely with OPT images (bottom panel).

**Supplemental Figure 2 Plasma triglyceride (A) and total cholesterol levels (B) were not affected by castration or vascular androgen receptor ablation. (**Tested by one-way ANOVA. WT=wild type litter mates carrying floxed-AR; SM-ARKO=AR ablated in SMC, VE-ARKO=AR ablated in EC, SM/VE-ARKO=AR ablated in both EC and SMC. n=6-13)

**Supplemental Figure 3 Vascular specific AR ablation has no impact on compliance in (A) femoral or (B) mesenteric arteries**. (WT=wild type litter mates carrying floxed-AR; SM-ARKO=AR ablated in SMC, VE-ARKO=AR ablated in EC, SM/VE-ARKO=AR ablated in both EC and SMC. n=11-16).

**Supplemental Figure 4. Influence of vascular specific androgen receptor (AR) ablation on femoral (A) and mesenteric (B) artery function**. Vascular ARKO did not alter KPSS-induced contraction (A(i); B(i)) or sodium nitroprusside (SNP)-induced relaxation (A(ii); B(ii)). * p<0.05, ** p<0.01 vs corresponding WT concentration; two-way ANOVA plus Bonferroni post-hoc test. (WT=wild type litter mates carrying floxed-AR; SM-ARKO=AR ablated in SMC, VE-ARKO=AR ablated in EC, SM/VE-ARKO=AR ablated in both EC and SMC. n=7-9).

**Supplemental Figure 5. The impact of castration on body weight change following surgery.** Body weight in castrated (Cas) mice dropped, compared with sham-operated controls following surgery. n=10-11, * p<0.05, **P<0.01 versus corresponding time point, by Student’s t-test.

**Supplemental Figure 6. The impact of androgen receptor (AR) deletion on body weight change following surgery.** Vascular AR ablation did not affect body weight change after arterial injury compared with wild type (WT), by one-way ANOVA. (WT=wild type litter mates carrying floxed-AR; SM-ARKO=AR ablated in SMC, VE-ARKO=AR ablated in EC, SM/VE-ARKO=AR ablated in both EC and SMC. n=7-14.

**References**

1. Welsh M, Saunders PT, Atanassova N, Sharpe RM, Smith LB. Androgen action via testicular peritubular myoid cells is essential for male fertility. *Faseb J* 2009;**23**:4218-4230.

2. O'Hara L, Smith LB. Androgen receptor signalling in Vascular Endothelial cells is dispensable for spermatogenesis and male fertility. *BMC Res Notes* 2012;**5**:16.

3. Kisanuki YY, Hammer RE, Miyazaki J, Williams SC, Richardson JA, Yanagisawa M. Tie2-Cre transgenic mice: a new model for endothelial cell-lineage analysis in vivo. *Dev Biol* 2001;**230**:230-242.

4. Holtwick R, Gotthardt M, Skryabin B, Steinmetz M, Potthast R, Zetsche B*, et al.* Smooth muscle-selective deletion of guanylyl cyclase-A prevents the acute but not chronic effects of ANP on blood pressure. *Proc Natl Acad Sci U S A* 2002;**99**:7142-7147.

5. Kobayashi M, Inoue K, Warabi E, Minami T, Kodama T. A simple method of isolating mouse aortic endothelial cells. *J Atheroscler Thromb* 2005;**12**:138-142.

6. Wu J, Wadsworth RM, Kennedy S. Inhibition of inducible nitric oxide synthase promotes vein graft neoadventitial inflammation and remodelling. *J Vasc Res* 2011;**48**:141-149.

7. Jones RD, Morice AH, Emery CJ. Effects of perinatal exposure to hypoxia upon the pulmonary circulation of the adult rat. *Physiol Res* 2004;**53**:11-17.

8. Sata M, Maejima Y, Adachi F, Fukino K, Saiura A, Sugiura S*, et al.* A mouse model of vascular injury that induces rapid onset of medial cell apoptosis followed by reproducible neointimal hyperplasia. *J Mol Cell Cardiol* 2000;**32**:2097-2104.

9. Kirkby NS, Low L, Seckl JR, Walker BR, Webb DJ, Hadoke PW. Quantitative 3-dimensional imaging of murine neointimal and atherosclerotic lesions by optical projection tomography. *PLoS One* 2011;**6**:e16906.


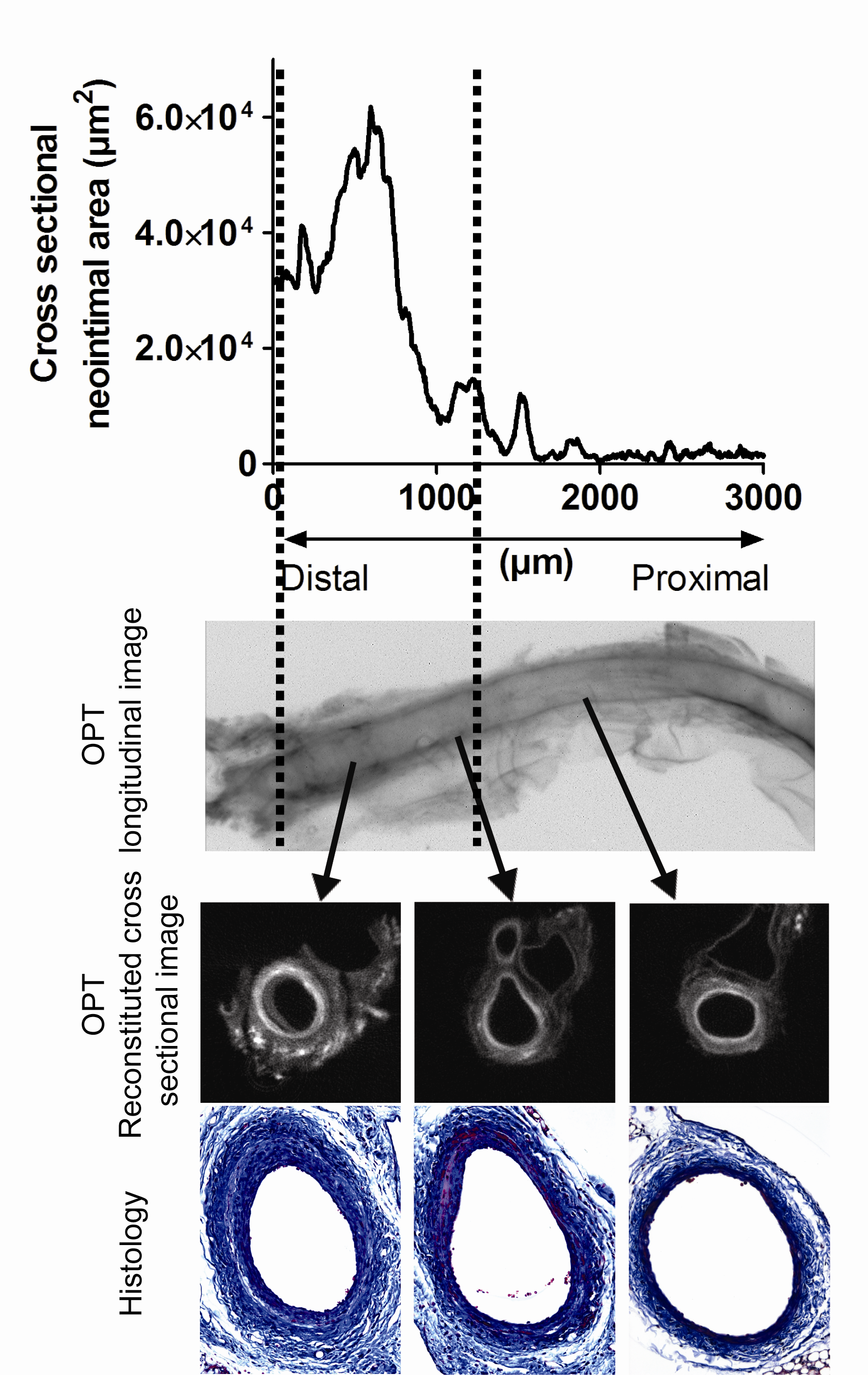


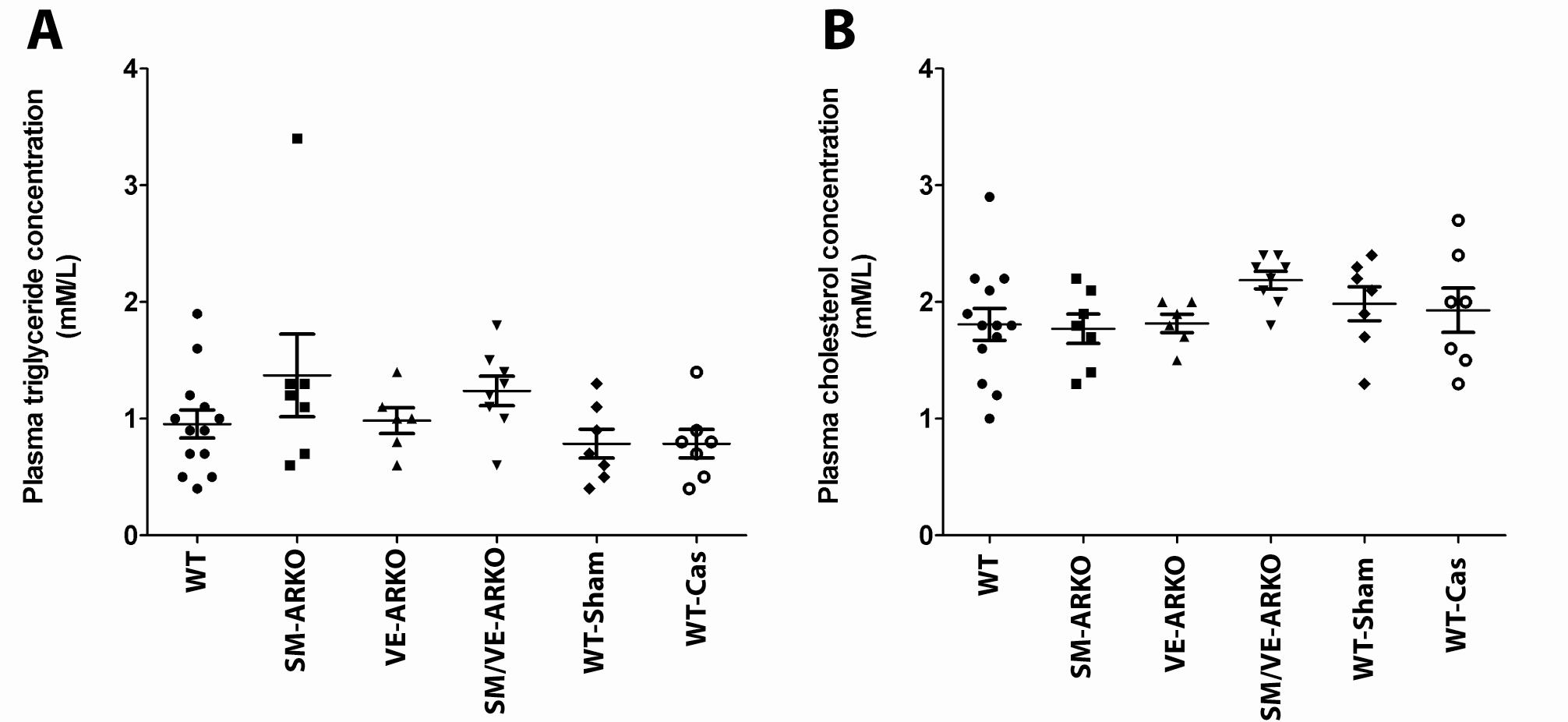


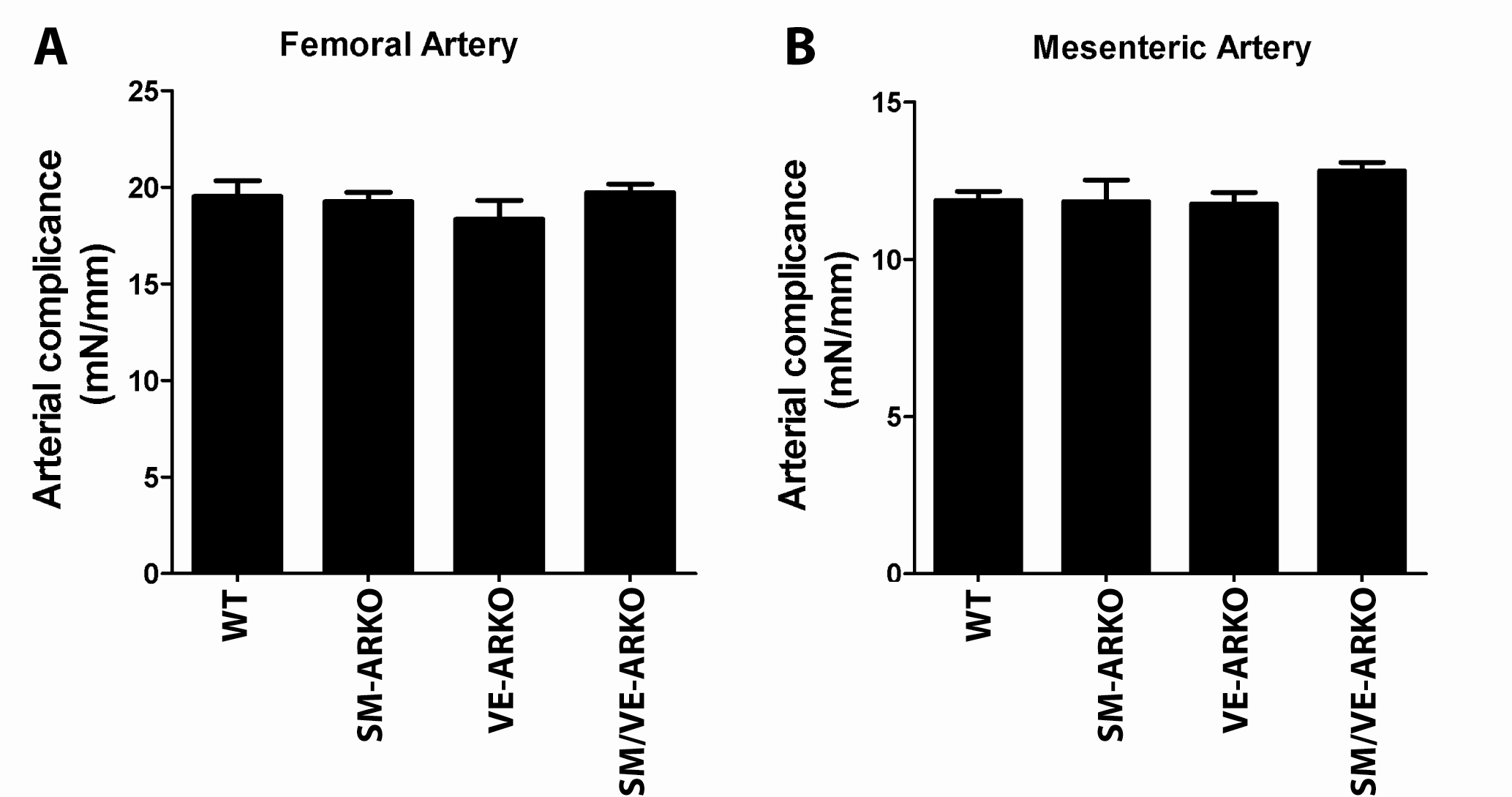


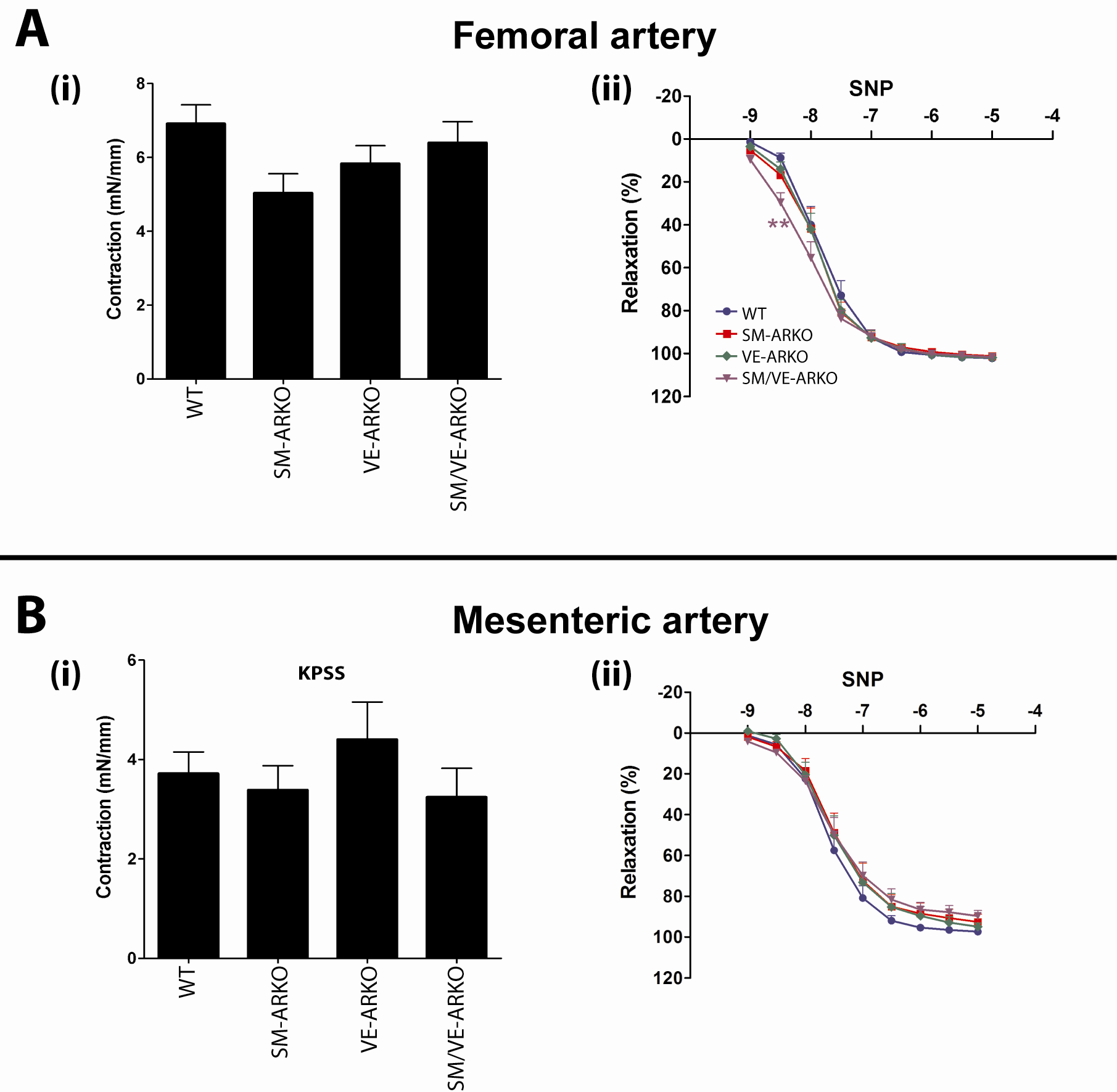


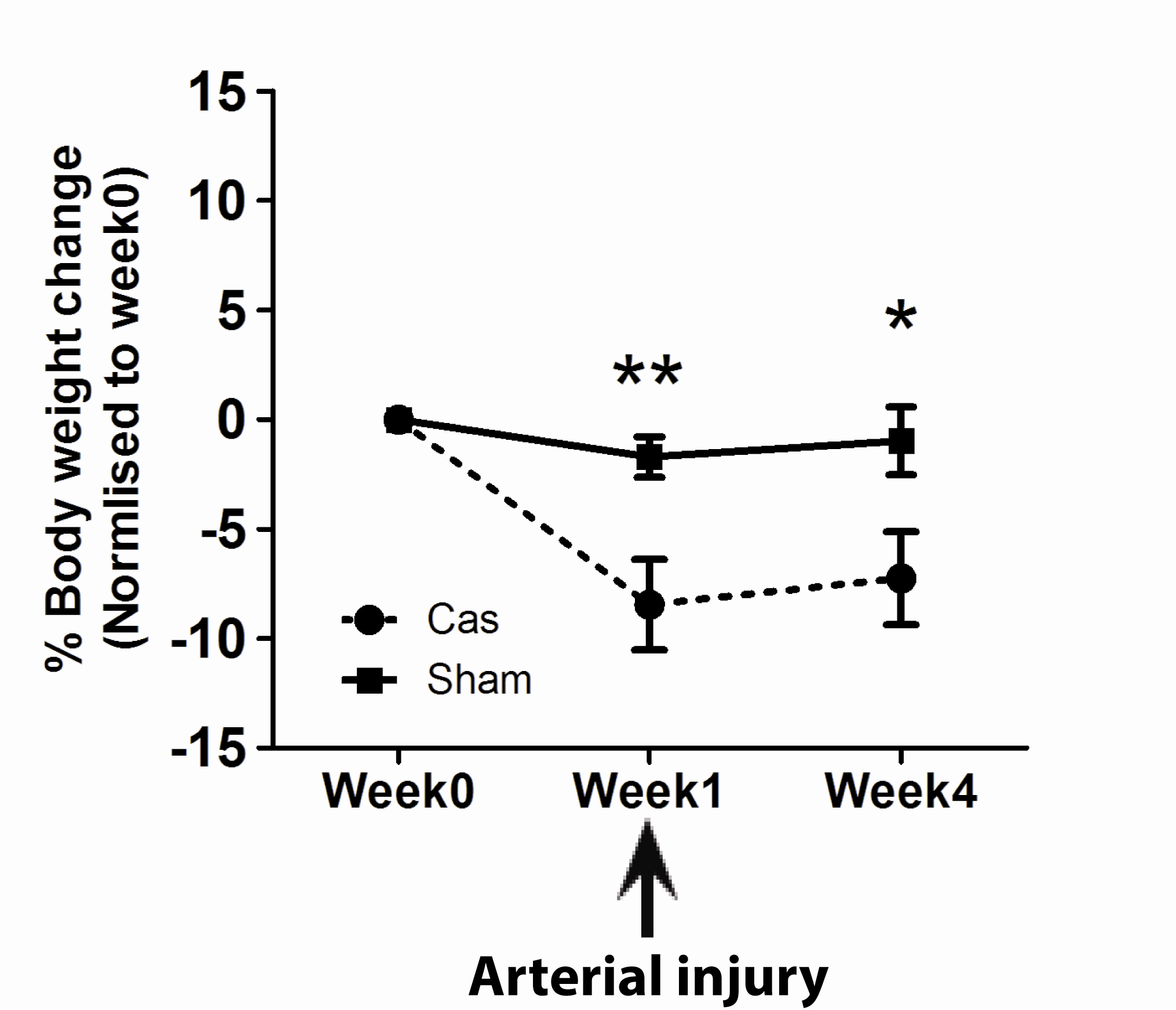


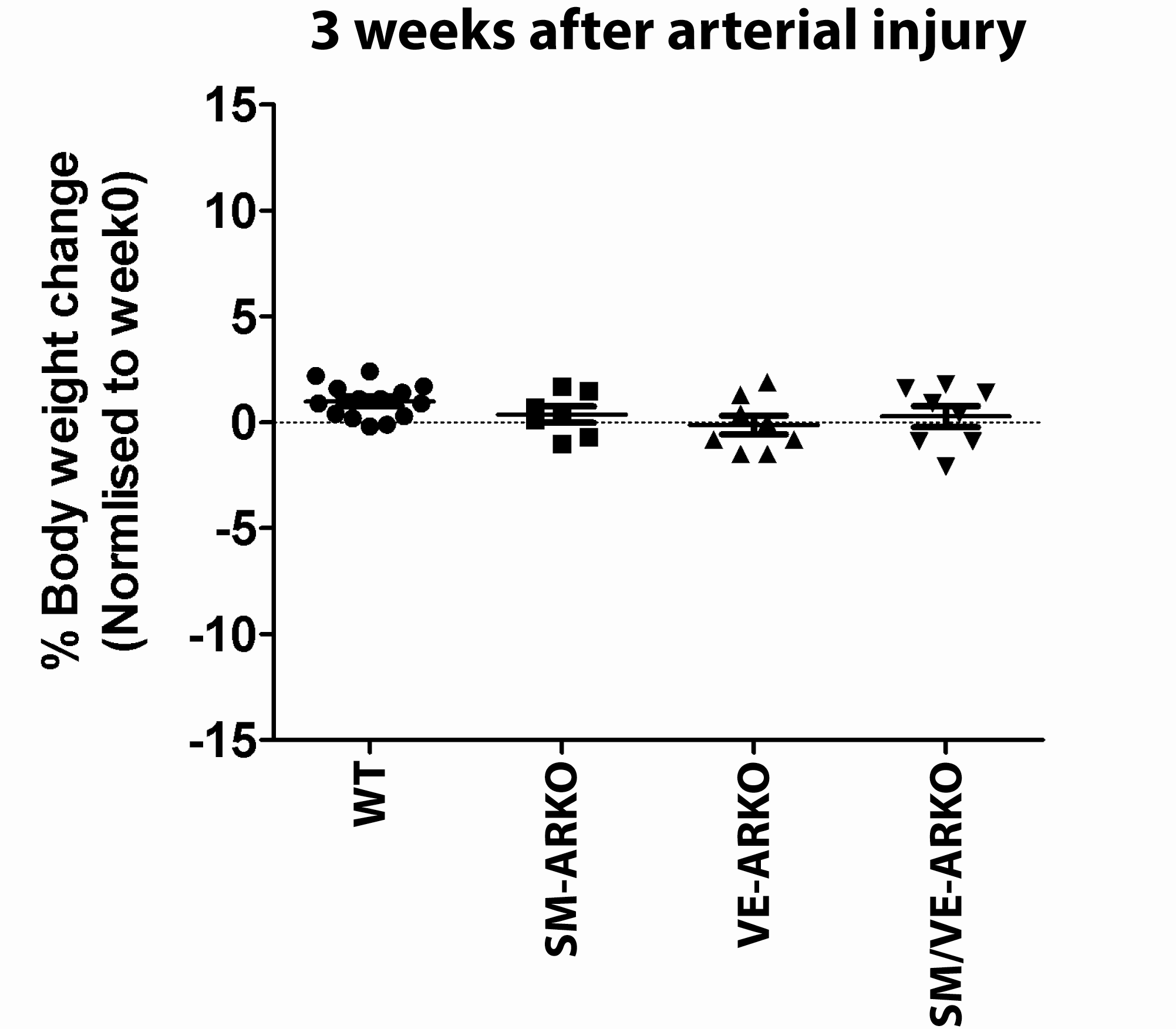

Supplement: Supplementary Data [file supp_cvu142_cvu142supp.doc]
